# Evaluation of Immunoprotective Effects of DNA Vaccine Based on *Eimeria maxima* EF-1α Antigen and Chicken XCL1 Chemokine

**DOI:** 10.3390/ani16071108

**Published:** 2026-04-03

**Authors:** Xiao-Feng Lin, Xi-Ge Wang, Chang-Sheng Fu, Zhong-Sheng Zhang, Hai-Yan Wu, Pan-Pan Guo, Deng-Feng Wang, Lei Wang, Yu-Tong Yan, Guang-Wen Yin

**Affiliations:** College of Animal Sciences, Fujian Agriculture and Forestry University, Fuzhou 350002, China; 19959156561@163.com (X.-F.L.); wxg210914@163.com (X.-G.W.); fuchangsheng2001@163.com (C.-S.F.); zzssamsung@163.com (Z.-S.Z.); wuhaiyan_linda@fafu.edu.cn (H.-Y.W.);

**Keywords:** *Eimeria maxima*, chicken, DNA vaccine, EmEF1α, ChXCL1

## Abstract

The moderate pathogenicity coupled with high host susceptibility of *Eimeria maxima* has precipitated substantial economic losses in the poultry industry. Addressing challenges such as emerging drug resistance underscores the imperative for innovative vaccine strategies. This study developed a novel DNA vaccine to solve this challenge by fusing *E. maxima* elongation factor-1α (EmEF1α) with chicken chemokine XCL1 (ChXCL1) in the pVAX1 vector. The recombinant plasmid, designated pVAX1-ChXCL1-EmEF1α, was successfully constructed and confirmed to express the ChXCL1-EmEF1α fusion protein in vitro. Immunization of chickens with this DNA vaccine elicited a robust and balanced immune response, characterized by significantly increased proportions of CD4^+^ (11.76%) and CD8^+^ (5.58%) T lymphocytes, elevated levels of Th1-associated cytokines (IFN-γ and IL-12), and strong antigen-specific IgG and IgA antibody responses. Following experimental challenge with *E. maxima*, vaccinated birds exhibited substantial protection: a 66.4% reduction in oocyst shedding, a 71.7% improvement in relative weight gain, marked attenuation of intestinal lesions, and an anticoccidial index (ACI) of 170. These findings demonstrate that the ChXCL1-EmEF1α DNA vaccine effectively enhances both cellular and humoral immunity. Collectively, this study validates ChXCL1 as a potent molecular adjuvant and establishes the “antigen–adjuvant” fusion DNA platform as a promising strategy for developing next-generation vaccines against avian coccidiosis.

## Simple Summary

Chicken coccidiosis caused by apicomplexan parasites of the genus *Eimeria* imposes substantial economic losses on the global poultry industry. To reduce the infection of *Eimeria maxima*, we developed a novel DNA vaccine comprising a fusion construct of the parasite-derived antigen EmEF1α and the chicken chemokine immune modulator ChXCL1. This fusion enhanced immunity, increasing CD4^+^ and CD8^+^ T cells and protective antibodies. Following experimental challenge with *E. maxima*, vaccinated birds exhibited robust clinical protection: intestinal lesions were reduced, weight gain increased by 71.7%, and oocyst shedding decreased by 66.4%. This study establishes an improved DNA vaccine strategy for protecting poultry health against coccidiosis.

## 1. Introduction

Avian coccidiosis represents a major constraint to poultry production, resulting in estimated annual economic losses of approximately $13 billion all over the world [1]. The seven *Eimeria* species show that infected chickens display distinct biological characteristics and trigger a highly destructive cycle within the intestinal epithelium. Parasite replication leads to villous atrophy and fusion and also significantly reduces the intestine’s absorptive surface area. As a result, digestive enzyme activity is suppressed, the expression of key nutrient transporters is downregulated, and the ecological equilibrium of the intestinal microbiota is disrupted. Collectively, these pathological alterations impair feed efficiency, induce diarrhea, and increase susceptibility to secondary bacterial infections—potentially culminating in mortality [2,3,4,5]. Disease management is currently dependent on drugs and live vaccines, but widespread drug resistance and incomplete vaccine protection limit their efficacy [6]. Therefore, developing novel strategies is essential for sustainable disease management, such as next-generation vaccines targeting new antigens.

Elongation factor-1α (EF1α) functions as a protective antigen that becomes accessible to the host immune system during coccidial invasion, thereby triggering immune responses capable of inhibiting infection. Early study uses the monoclonal antibody 6D-12-G10 localized EF1α to the apical complex of *Eimeria necatrix* and demonstrated its capacity to inhibit sporozoite invasion of CD8^+^ T cells [7]. EF1α is expressed in both sporozoite and schizont stages of *E. tenella*, and anti-EF1α polyclonal antibodies have been shown to inhibit approximately 22% of sporozoite invasion in vitro, underscoring its essential role in parasite development [8]. Owing to its high degree of conservation, recombinant EF1α-base subunit vaccines have been found to significantly elevate antigen-specific antibody titers and reduce oocyst shedding in experimental infections with *E. maxima* and *E. tenella* [9]. Furthermore, the protective efficacy of EF1α is significantly enhanced upon co-administration with immunomodulatory agents, including IL-7, the cNK2 peptide and ChXCL1, which result in attenuated intestinal lesions, modulation of cytokine expression, and improved weight gain [10,11,12].

Lymphocyte chemotactic protein-1 (XCL1) exists in two structurally and functionally distinct conformations: an α–β monomeric form that mediates signaling through the XCR1 receptor and a β-dimer form that binds glycosaminoglycans to exert antimicrobial activity [13]. XCL1 establishes a targeted delivery axis by specifically binding to the XCR1 receptor that is expressed on dendritic cells, enabling precise antigen delivery, facilitating efficient cross-presentation, and robustly activating antigen-specific CD8^+^ T cell responses, thereby strengthening the host’s defense against intracellular pathogens [14]. XCL1 can promote dendritic cell migration and drives the generation of durable, antigen-specific memory T cells [15]. In vaccine development, the fusion of XCL1 with influenza hemagglutinin (HA) or the SARS-CoV-2 spike (S) protein enhances Th1-biased immunity and improves protective efficacy [16,17]. Similarly, fusion with tumor-associated antigen augments the immunogenicity and antitumor activity of DNA vaccines [18]. Chicken XCL1 (ChXCL1) is a secreted chemokine whose recombinant vaccines have been shown to significantly reduce oocyst shedding and enhance weight gain in chickens infected with *Eimeria* spp. [11,19,20]. The XCL1–XCR1 signaling axis on chicken conventional type 1 dendritic cells (cDC1s) provides a mechanistic foundation for the development of next-generation coccidiosis vaccines [21].

Given the immunogenic potential of EF1α, a novel DNA vaccine was developed by fusing *Eimeria maxima* EF1α (EmEF1α) with the chemokine ChXCL1 in the pVAX1 vector. This fusion construct was shown to significantly boost immunogenicity and provide effective protection against a challenge with *E. maxima*. The “adjuvant-antigen” fusion strategy is validated and a scalable platform for developing multivalent nucleic acid vaccines against coccidiosis and other avian diseases is established by this study.

## 2. Materials and Methods

### 2.1. Animals and Parasites

One-day-old male chicks were obtained from Fuzhou Nongken Breeding Poultry Co., Ltd. (Fuzhou, China) and housed in a strictly disinfected animal facility, with free access to water and feed. Prior to the commencement of experiment, fecal samples were collected and examined to confirm the absence of *E. maxima* infection.

*E. maxima* oocysts (Beijing strain) were provided by Prof. Suo Xun’s laboratory at China Agricultural University. To ensure oocyst viability and maintain the parasite strain, a standard propagation protocol was followed: three-day-old chicks were orally inoculated with 1 × 10^4^ oocysts per bird. Fresh fecal samples were collected between days 6 and 9 post-infection, and unsporulated oocysts were isolated using saturated salt flotation. The recovered oocysts were then incubated under appropriate conditions to induce sporulation. Sporulated oocysts were identified and quantified by light microscopy and validated using established molecular methods [22]. Only batches exhibiting a sporulation rate >85% were selected for subsequent challenge experiments.

### 2.2. Cloning and Plasmid Construction of ChXCL1, EmEF1α, and ChXCL1-EmEF1α Genes

Specific primers for amplifying *ChXCL1*, *EmEF1α*, and the *ChXCL1-EmEF1α* fusion gene were designed based on the GenBank sequences NP_990377 and XP_013336300.1, and commercially synthesized by Fuzhou Shangya Biotechnology Co., Ltd. (Fuzhou, China) (Table 1). Target DNA fragments were amplified via polymerase chain reaction (PCR). Following the manufacturer’s protocol, PCR products were purified using a gel extraction kit and subsequently ligated into the pEASY^®^-Blunt Simple Cloning Vector (TransGen Biotech, Beijing, China). Recombinant clones were selected for plasmid extraction and sequenced to verify the correct insertion of each gene fragment.

For subcloning into the eukaryotic expression vector, the pEASY^®^-ChXCL1 and pEASY^®^-ChXCL1-EmEF1α and pVAX1 (Sangon Biotech, Shanghai, China) were individually digested with Afl II and Xho I (Takara Bio Inc., Kusatsu, Shiga, Japan), whereas pEASY^®^-EmEF1α and pVAX1 were digested with EcoR I (Takara Bio Inc., Kusatsu, Shiga, Japan) and Xho I. Following gel purification, the respective fragments were directionally ligated into the pVAX1 vector using T_4_ DNA ligase (TransGen Biotech Co., Ltd., Beijing, China), yielding the constructs pVAX1-ChXCL1, pVAX1-EmEF1α, and pVAX1-ChXCL1-EmEF1α-all of which were confirmed by Sanger sequencing.

### 2.3. The Construction of DNA Vaccines pVAX1-ChXCL1, pVAX1-EmEF1α, and pVAX1-ChXCL1-EmEF1α

Transient transfection of the constructed eukaryotic plasmids was carried out in COS-7 cells, following an established protocol with minor modification [23]. Transfection complex was prepared using a lipid-based reagent. Briefly, plasmids (1 µg; pVAX1-ChXCL1, pVAX1-EmEF1α, pVAX1-ChXCL1-EmEF1α, or the empty pVAX1 vector control) and TransIntro™ EL Transfection Reagent (2 µL; TransGen Biotech Co., Ltd., Beijing, China) were separately diluted in 50 µL aliquots of opti-MEM^®^ medium (Gibco, Thermo Fisher Scientific, Waltham, MA, USA). Both dilutions were incubated at room temperature for 5 min prior to combination. The DNA-lipid mixture was then incubated for an additional 20 min at room temperature to allow complex formation. The COS-7 cell line, maintained at the College of Animal Science, Fujian Agriculture and Forestry University, supports highly efficient transient expression of exogenous genes and is widely employed for recombinant protein production and functional characterization studies. For transfection, cells were seeded into 6-well plates at a density of 3 × 10^5^ cells per well and cultured in high-glucose DMEM (Gibco, Thermo Fisher Scientific, Waltham, MA, USA) supplemented with 10% fetal bovine serum (FBS; ZETA Life Biotechnology Co., Ltd., Menlo Park, CA, USA) at 37 °C under a humidified atmosphere containing 5% CO_2_. When cells reached approximately 70% confluence, the culture medium was replaced, and transfection complexes were added. After a 6 h incubation, the medium was refreshed, and cells were further cultured for an additional 24 h.

To confirm protein expression, transfected cells were processed for immunofluorescence staining analysis [24]. Cells were fixed, permeabilized, and blocked with 5% bovine serum albumin (BSA). Subsequently, they were incubated overnight at 4 °C with a mouse anti-His tag primary antibody (Zen Bioscience Co., Ltd., Chengdu, China). Following extensive washing, the cells were incubated for 1 h at room temperature in dark with a FITC-conjugated goat anti-mouse IgG (H + L) secondary antibody (Zen Bioscience Co., Ltd., Chengdu, China). Finally, cells were washed, and fluorescence signals were visualized and documented using an inverted fluorescence microscope (Nikon, Tokyo, Japan).

### 2.4. Reverse Transcription-PCR (RT-PCR)

Forty-eight hours after transfection with the respective plasmids, total RNA was extracted from COS-7 cells using the TransZol Up kit (TransGen Biotech, Beijing, China) following the manufacturer’s instruction. To confirm transcription, cDNA was synthesized and subjected to RT-PCR using gene-specific primers listed in Table 1. The resulting amplification products were separated and visualized on a 1% agarose gel stained with ethidium bromide.

### 2.5. Western Blot

Protein expression was assessed by Western blot analysis after 48 h post-transfection. Cells were harvested and lysed, and the resulting lysates were resolved by SDS–PAGE, followed by electrophoretic transfer onto a PVDF membrane (Millipore, Burlington, MA, USA). The membrane was incubated overnight at 4 °C with a mouse anti-His tag primary antibody (1:5000 dilution), followed by incubation with an HRP-conjugated goat anti-mouse IgG secondary antibody (1:5000 dilution; Zen Bioscience Co., Ltd., Chengdu, China) for 1 h at room temperature. Protein bands were detected using an enhanced chemiluminescence (ECL) substrate kit (ZETA Life Biotechnology Co., Ltd., Menlo Park, CA, USA) and imaged.

### 2.6. Animal Experiment

Chickens with comparable body weights at 4 days of age were randomly assigned to six experimental groups (*n* = 15): (1) unvaccinated and unchallenged control group (using PBS instead of vaccine; “uu” group); (2) unvaccinated and challenged control group (using PBS instead of vaccine; “uc” group); (3) pVAX1 empty vector control group; (4) pVAX1-ChXCL1 group; (5) pVAX1-EmEF1α group; and (6) pVAX1-ChXCL1-EmEF1α group. Chickens in groups 3 to 6 received three intramuscular immunizations (100 µg plasmid per dose) in the thigh muscle at 4, 18, and 32 days of age. Concurrently, chickens in the unvaccinated control groups (uu and uc) received 200 µL of PBS (Solarbio, Beijing, China) via the same route at each corresponding time point. Fourteen days after the final immunization (at 46 days of age), all chickens were orally challenged with 5.0 × 10^4^ sporulated *E. maxima* oocysts to evaluate vaccine efficacy except the uu group. Meanwhile, all chickens were humanely euthanized via intravenous injection of an overdose of air at 55 days of age.

### 2.7. Analysis of CD4^+^ and CD8^+^ T Cell Populations by Flow Cytometry

At 14 days post-third immunization (Day 46), whole blood was collected from the wing vein, and peripheral blood mononuclear cells (PBMCs) were isolated using a commercial lymphocyte separation kit (Solarbio, Beijing, China). The isolated cells were washed and subsequently resuspended in 100 µL of PBS. For immunophenotypic analysis, cells were incubated for 1 h at room temperature in dark with a fluorescent antibody cocktail containing 1 µL PE-conjugated anti-CD3, 0.5 µL AF647-conjugated anti-CD4, and 1 µL FITC-conjugated anti-CD8a (Southern Biotechnology Associates, Birmingham, AL, USA). Following incubation, cells were washed and resuspended in PBS and acquired on a NovoCyte flow cytometer (ACEA Biosciences, Inc., San Diego, CA, USA). Data were analyzed using NovoExpress^®^ software (version 1.5.0) to determine the proportions of CD3^+^CD4^+^ and CD3^+^CD8^+^ T lymphocyte subsets.

### 2.8. Serum Anti-EF1α Antibody Detection

Serum samples were collected from the wing vein on day 14 following each immunization (Day 18, 32, and 46). Following a 1 h incubation at room temperature, samples were centrifuged to separate serum, which was then aliquoted and stored at −80 °C until analysis. Levels of anti-EmEF1α IgG and IgA antibodies were quantified by enzyme-linked immunosorbent assay (ELISA) [10]. Briefly, 96-well plates were coated overnight at 4 °C with purified recombinant EmEF1α antigen (4 µg/mL) dissolved in coating buffer. Subsequently, the plates were washed three times with phosphate-buffered saline containing 0.05% Tween-20 (PBST) and blocked overnight at 4 °C with 5% (*w*/*v*) skimmed milk. Serum samples, serving as primary antibodies, were diluted 1:100 in 2% (*w*/*v*) skimmed milk and incubated for 1 h at 37 °C. After washing, HRP-conjugated goat anti-chicken IgG or IgA secondary antibody (Bethyl Laboratories, Inc., Montgomery, TX, USA; 1:10,000 dilution) was incubated for 1 h at 37 °C. Following a final wash, TMB substrate solution (Solarbio, Beijing, China) was added and allowed to develop in dark at room temperature for 15 min. The reaction was terminated with stop solution, and absorbance was measured at 450 nm. Antibody titers were determined by serial two-fold dilutions (1:100, 1:200, 1:400, 1:800, 1:1600, 1:3200, 1:6400, and 1:12,800) of serum collected 14 days after the third immunization; all subsequent ELISA steps were performed identically to those described above for IgG and IgA detection.

### 2.9. Serum Cytokine Profiling

Serum concentration of chicken IL-4, IL-10, IL-12, and IFN-γ was measured using commercial ELISA kits (CusaBio, Wuhan, China) according to manufacturer instructions, respectively [25]. Serum was collected as a sample for cytokine analysis on day 14 (Day 46) post-third immunization. Briefly, a standard curve was first generated using the provided recombinant cytokine standards. For sample analysis, 50 µL of appropriately diluted serum was added to each well of a pre-coated 96-well plate. Subsequently, 50 µL of enzyme-conjugated antibody and 50 µL of detection antibody were added to each well, followed by incubation at 37 °C for 1 h. After washing to remove unbound components, 50 µL each of chromogen solution A and B were added to the wells. The plate was gently mixed and incubated at 37 °C in dark for 15 min to allow color development. The reaction was then stopped by adding 50 µL of stop solution per well, and the optical density at 450 nm was immediately measured using a microplate reader.

### 2.10. Immunoprotective Evaluation In Vivo

The immunoprotective efficacy of the DNA vaccine was assessed using a comprehensive set of parameters, including weight gain, jejunal lesion severity, oocyst shedding, and the Anticoccidial Index (ACI). Body weights were recorded at two time points: pre-challenge (Day 46, prior to *E. maxima* challenge) and on day 9 post-challenge (Day 55). The average weight gain was calculated as follows: [(Final total group body weight) − (Initial total group body weight)]/(Number of chickens per group). Relative Weight Gain (RWG) was then determined as: RWG (%) = [(Average weight gain of the vaccinated or unvaccinated and challenged control group)/(Average weight gain of the unvaccinated and unchallenged control group)] × 100% [26]. On day 5 of post-challenge, chickens were fasted. The following day (Day 52), they were humanely euthanized, and jejunal lesions were assessed and scored using a standardized 0–4 grading scale [27]. Oocyst shedding was quantified using the McMaster technique. Briefly, 2 g of fecal sample was suspended in 58 mL of saturated sodium nitrate solution, filtered through a standard sieve, and the resulting suspension was loaded into McMaster counting chambers. After a 5 min settling period, oocysts per gram (OPG) of feces were calculated using the formula: (average oocyst count per chamber/0.15) × 30, where 0.15 accounts for the volume (in mL) of fecal suspension loaded into each chamber, and 30 represents the overall dilution factor [28]. Simultaneously, calculate the reduction rate of oocysts as follows: [average oocyst count of the challenge control group] − [average oocyst count of the vaccination group]/[average oocyst count of the challenge control group] × 100% [29]. The overall protective efficacy was synthesized into the ACI, calculated with the formula: ACI = [(Survival Rate + RWG) × 100] − (Lesion Score+ Oocyst Index) [30]. The ACI serves as a key composite indicator, with its value interpreted as follows: ≥180 indicates excellent efficacy; 160–179, good efficacy; 120–159, fair efficacy; and <120, insufficient protective efficacy [31].

### 2.11. Statistical Analysis

Data are presented as mean ± standard deviation (SD). Statistical significance was determined by one-way ANOVA followed by Tukey’s post hoc test using SPSS software (version 25.0, IBM, Armonk, NY, USA). Graphical representations were generated using GraphPad Prism 9.5 (GraphPad Software). Each experiment was repeated with at least three biological replicates. Differences were considered statistically significant at *p* < 0.05.

## 3. Results

### 3.1. Gene Cloning and Plasmid Construction 

Utilizing the existing plasmid pET28a-ChXCL1-EmEF1α as a template, the *ChXCL1*, *EmEF1α*, and *ChXCL1-EmEF1α* fusion genes were amplified by PCR. Agarose gel electrophoresis confirmed the amplification, showing products corresponding of the expected sizes: 264 bp for *ChXCL1*, 1389 bp for *EmEF1α*, and 1614 bp for the *ChXCL1-EmEF1α* fusion fragment (Figure S1A–C). Each PCR-amplified fragment was cloned into the pVAX1 vector and validated by restriction enzyme digestion and Sanger sequencing (Figure S1D), confirming the correct construction of the pVAX1-ChXCL1, pVAX1-EmEF1α, and pVAX1-ChXCL1-EmEF1α plasmids.

### 3.2. Eukaryotic Expression of ChXCL1, EmEF1α, and ChXCL1-EmEF1α DNA Plasmid

**Figure 1:**
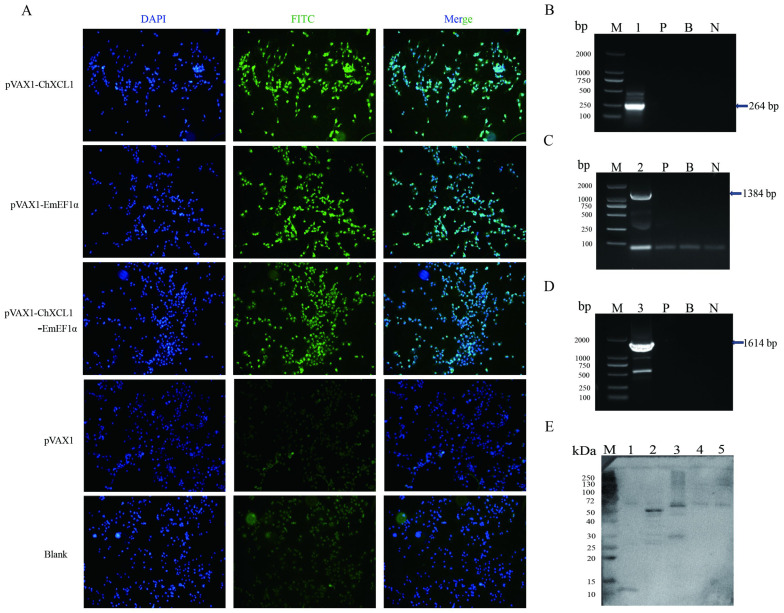
Eukaryotic expression of ChXCL1, EmEF1α, and ChXCL1-EmEF1α DNA plasmids. (**A**): Fluorescence image under the microscope (×100). (**B**–**D**): RT-PCR assay of *ChXCL1*, *EmEF1α*, and *ChXCL1-EmEF1α* gene. Lane M: Trans2K DNA Marker; lane 1: *ChXCL1* gene; lane 2: *EmEF1α* gene; lane 3: *ChXCL1-EmEF1α* gene; lane P: pVAX1 group; lane B: blank group; lane N: negative control group. (**E**): Western blot analysis of ChXCL1, EmEF1α and ChXCL1-EmEF1α gene expression in COS-7 cells. Lane M: Standard protein marker (10–250 kDa); lane 1: ChXCL1; lane 2: EmEF1α; lane 3: ChXCL1-EmEF1α; lane 4: pVAX1 group; lane 5: blank group.

The expression of the constructed plasmids was systematically assessed using complementary analytical approaches. Green fluorescence was observed in COS-7 cells transfected with pVAX1-ChXCL1, pVAX1-EmEF1α, or pVAX1-ChXCL1-EmEF1α (Figure 1A). RT-PCR analysis revealed the presence of the corresponding specific mRNA transcripts (Figure 1B–D), and Western blot analysis identified bands at approximately 10 kDa, 56 kDa, and 65 kDa, consistent with the predicted molecular weights of the ChXCL1, EmEF1α, and ChXCL1-EmEF1α fusion proteins, respectively (Figure 1E). The concordance of these results across three independent methodologies conclusively confirms the successful in vitro expression of all three recombinant constructs in COS-7 cells.

### 3.3. Flow Cytometric Analysis of CD4^+^ and CD8^+^ T Cell Populations

**Figure 2:**
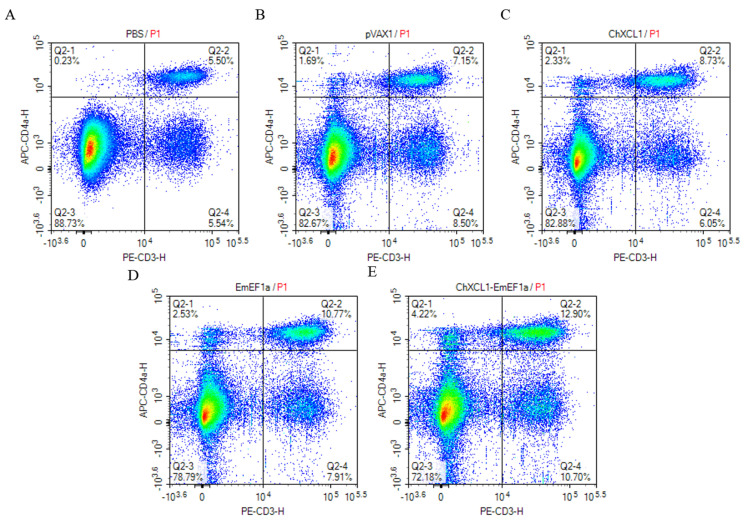
The investigation of CD3^+^ CD4^+^ lymphocyte subsets post-third immunization. (**A**): PBS group; (**B**): pVAX1 group; (**C**): pVAX1-ChXCL1 group; (**D**): pVAX1-EmEF1α group; (**E**): pVAX1-ChXCL1-EmEF1α group. The density plots show the correlation between FITC-CD4a and PE-CD3. The color scale represents cell density, with blue indicating low density and red indicating the highest density of cell events. The plots are divided into four quadrants (Q1–Q4). Q1 represents [CD4]^+^/[CD3]^−^ cells; Q2 represents double-positive cells; Q3 represents double-negative cells; and Q4 represents [CD4]^−^/[CD3]^+^ cells. The percentage of cells in each quadrant is indicated.

**Figure 3:**
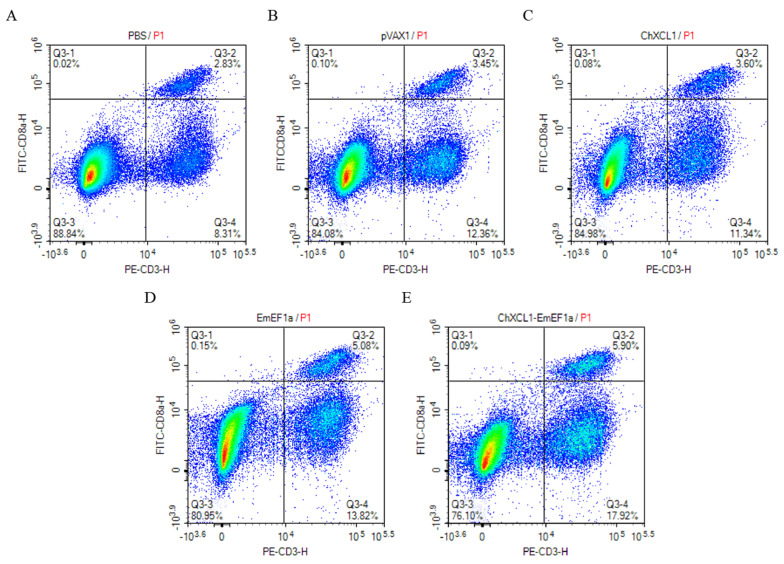
The investigation of CD3^+^ CD8^+^ lymphocyte subsets post-third immunization. (**A**): PBS group; (**B**): pVAX1 group; (**C**): pVAX1-ChXCL1 group; (**D**): pVAX1-EmEF1α group; (**E**): pVAX1-ChXCL1-EmEF1α group. The density plots show the correlation between FITC-CD8a and PE-CD3. The color scale represents cell density, with blue indicating low density and red indicating the highest density of cell events. The plots are divided into four quadrants (Q1–Q4). Q1 represents [CD8]^+^/[CD3]^−^ cells; Q2 represents double-positive cells; Q3 represents double-negative cells; and Q4 represents [CD8]^−^/[CD3]^+^ cells. The percentage of cells in each quadrant is indicated.

The proportions of CD4^+^ and CD8^+^ T lymphocytes in immunized chickens were assessed by flow cytometry (Figure 2 and Figure 3 and Table 2). All treatment groups exhibited a significant increase in the percentage of CD4^+^ T cells. and the pVAX1-ChXCL1-EmEF1α group exhibited the most notable increase at 11.76% (*p* < 0.05, Table 2). For CD8^+^ T cell responses, the pVAX1-EmEF1α (4.81%) and pVAX1-ChXCL1-EmEF1α (5.58%) groups had significantly higher proportions than the control and pVAX1-ChXCL1 (3.87%) groups (*p* < 0.05, Table 2). These findings suggest that the ChXCL1-EmEF1α fusion construct effectively enhances cellular immune responses by promoting the expansion of both CD4^+^ and CD8^+^ T lymphocyte subsets.

### 3.4. Serum Levels of Anti-EF1α Antibodies

**Figure 4:**
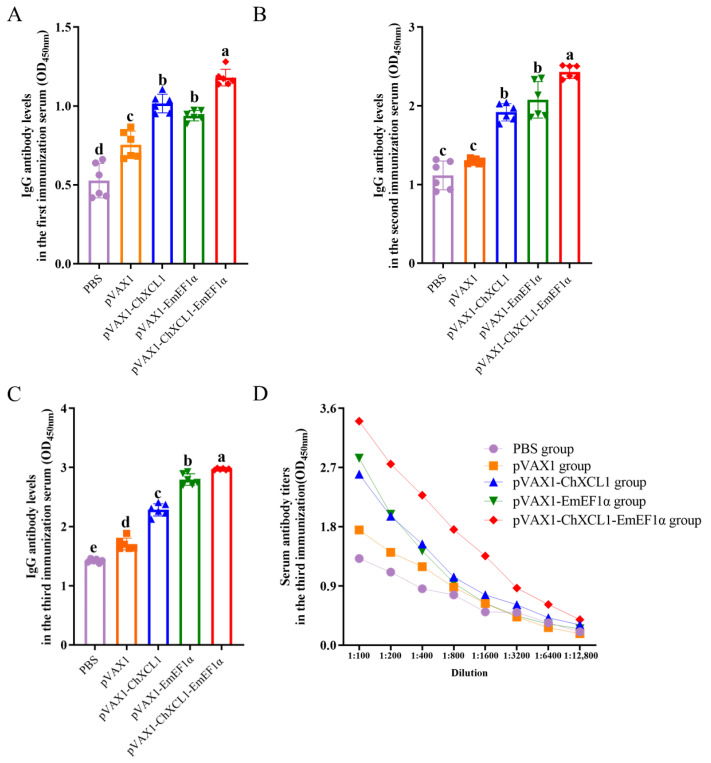
Evaluation of IgG antibody levels and titers in serum. (**A**): Serum IgG antibody levels in chickens after the first immunization. (**B**): Serum IgG antibody levels in chickens after the second immunization. (**C**): Serum IgG antibody levels in chickens after the third immunization. (**D**): Serum IgG antibody titers in chickens after the third immunization. In the same column, different letters indicate significant differences between groups (*p* < 0.05) (*n* = 6).

**Figure 5:**
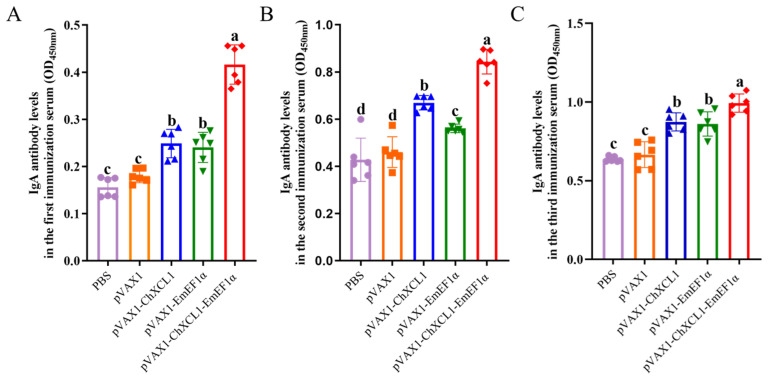
Evaluation of IgA antibody levels in serum. (**A**): Serum IgA antibody levels in chickens after the first immunization. (**B**): Serum IgA antibody levels in chickens after the second immunization. (**C**): Serum IgA antibody levels in chickens after the third immunization. In the same column, different letters indicate significant differences between groups (*p* < 0.05) (*n* = 6).

Serological analysis revealed that vaccination elicited a robust humoral immune response. Specifically, the pVAX1-ChXCL1-EmEF1α group exhibited significantly higher levels of anti-EF1α IgG and IgA antibodies as early as 14 days post-primary immunization compared with all other groups (*p* < 0.05). This superior antibody response was sustained following both booster immunizations, with this group consistently displaying the highest antibody levels throughout the entire experimental period. In contrast, no significant differences in IgG or IgA levels were observed between the PBS control group and the pVAX1 empty vector group (*p* > 0.05) (Figure 4A–C and Figure 5A–C). Furthermore, while antibody titers expectedly decreased with serial serum dilution, the pVAX1-ChXCL1-EmEF1α group retained a detectable and comparatively higher titer even at a high dilution of 1:6400, underscoring the potent and sustained antibody response induced by the fusion construct (Figure 4D). In conclusion, the pVAX1-ChXCL1-EmEF1α fusion construct effectively induces a strong and long-lasting antigen-specific antibody response, which has high levels and sustains serological reactivity.

### 3.5. Serum Cytokine Analysis of Immunized Chickens

**Figure 6:**
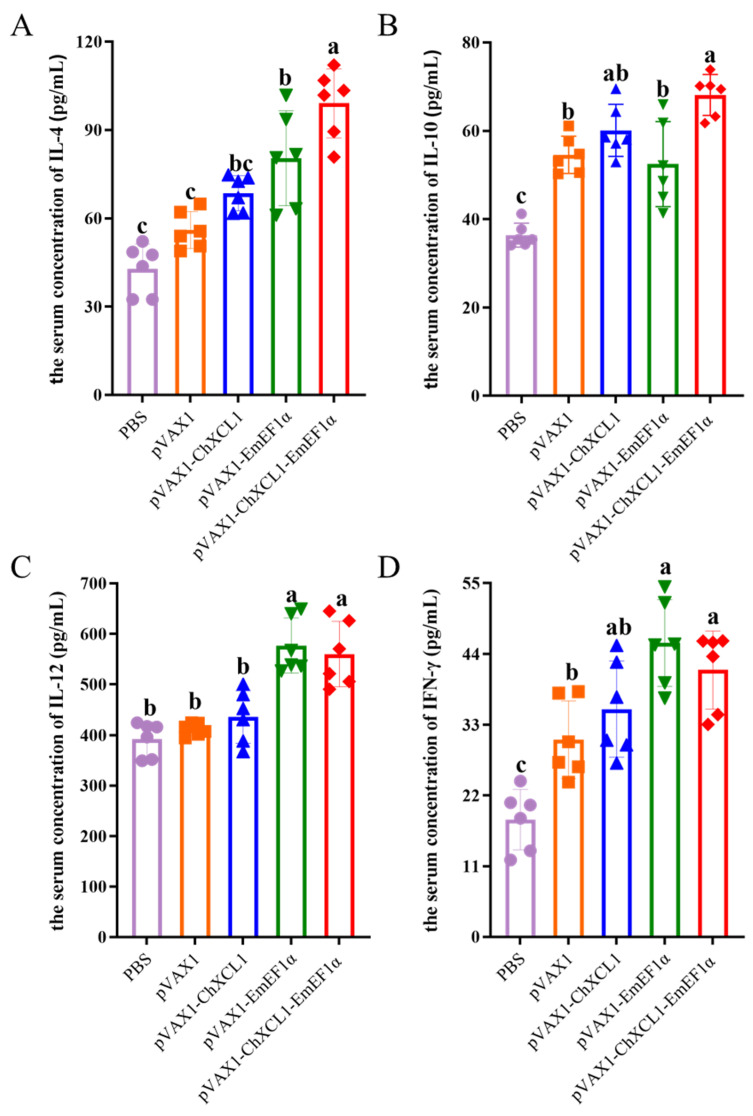
The concentration of cytokines IL-4, IL-10, IL-12, and IFN-γ in serum after the third immunization. (**A**): IL-4; (**B**): IL-10; (**C**): IL-12; (**D**): IFN-γ. In the same column, different letters indicate significant differences between groups (*p* < 0.05) (*n* = 6).

Serum concentrations of IL-4, IL-10, IL-12, and IFN-γ were quantified by ELISA (Figure 6). Statistical analysis revealed distinct cytokine production profiles across the vaccine groups. The pVAX1-ChXCL1-EmEF1α fusion vaccine group exhibited the highest IL-4 levels; modest increases were also observed in both the pVAX1-ChXCL1 and pVAX1-EmEF1α single-component groups. IL-10 levels were significantly elevated in the fusion vaccine group and the pVAX1-ChXCL1 group relative to all other groups (*p* < 0.05). With respect to Th1-associated cytokines, both the pVAX1-EmEF1α and the pVAX1-ChXCL1-EmEF1α fusion vaccine groups showed significantly higher IL-12 levels than the control group (*p* < 0.05). Notably, the pVAX1-EmEF1α single-component vaccine induced the most robust IFN-γ secretion-surpassing that elicited by either the fusion vaccine or the pVAX1-ChXCL1 group. Collectively, the pVAX1-ChXCL1-EmEF1α fusion vaccine induced a mixed Th1/Th2 cytokine profile (elevated IL-4, IL-10, IL-12), whereas the pVAX1-EmEF1α vaccine prompted a more polarized Th1 response dominated by IFN-γ.

### 3.6. In Vivo Immunoprotective of ChXCL1, EmEF1α, and ChXCL1-EmEF1α Construct

The protective immunity offered by the vaccine candidate was evaluated in chickens after three immunizations, followed by a challenge with *E. maxima*. Post-infection analysis of fecal oocyst shedding showed significant differences among treatment groups (*p* < 0.05, Table 3). The infected, unvaccinated and challenged control (uc) group and the group receiving the empty pVAX1 vector exhibited the highest oocyst output, indicating no protective immunity. In contrast, the pVAX1-ChXCL1-EmEF1α group shed the fewest oocysts, demonstrating a notable reduction in parasite replication. This finding was supported by gross jejunal lesion scoring. Although all challenged groups showed intestinal pathology, the most severe lesions were in the infection control and pVAX1 vector groups. Conversely, the pVAX1-ChXCL1-EmEF1α group exhibited the mildest pathological changes, suggesting that the vaccine-induced immunity effectively reduced infection-related tissue damage. Moreover, its relative weight gain rate post-challenge was the highest among infected groups, second only to the unchallenged healthy controls. Similarly, the ACI for this group was 170-ranking second overall, just below that of the unvaccinated and unchallenged control (uu) group (ACI = 200, representing the theoretical maximum protection achievable in the absence of both vaccination and *E. maxima* challenge), which indicated a moderate yet clinically relevant level of protection. Collectively, consistent improvements in oocyst shedding, intestinal lesion scores, weight gain, and the ACI collectively demonstrate that the EmEF1α-ChXCL1 DNA vaccine induces a robust immune response and confers substantial protection against *Eimeria* challenge.

## 4. Discussion

The development of vaccines against avian coccidiosis has witnessed progressive validation of DNA vaccine platform from initial proof of concept studies using pcDNA3-SO7 to more advanced and rationally designed constructs [32]. Notable examples include DNA vaccines encoding *E. tenella* fusion antigens (EtSAG16/22), which significantly reduced oocyst shedding and improved weight gain in challenged birds [23], and a multi-epitope DNA vaccine demonstrating broad-spectrum protection (ACI > 170) against multiple *Eimeria* species [33]. Building upon this well-established platform, our study advances the protective ChXCL1-EmEF1α antigen from its current subunit formulation into a DNA based delivery format. We aim to rigorously evaluate whether this modality enhances the immunogenic profile-particularly the induction of robust cellular immunity-and thereby improves protective efficacy against avian coccidiosis.

The fusion of ChXCL1 and EmEF1α synergistically enhances multi-level protective immunity against *E. maxima*. Flow cytometric analysis revealed a significant increase in the proportion of CD3^+^CD4^+^ T cells (12.90%) in the pVAX1-ChXCL1-EmEF1α-immunized group, confirming that ChXCL1 effectively expands antigen-specific CD4^+^ T-cell for sustained protective immunity [34,35,36]. This vaccine simultaneously induced robust Th1-polarized immunity, characterized by markedly elevated secretion of IFN-γ and IL-12 to exert direct anti-parasitic effects and potentiate cytotoxic T-cell function [37,38]. Consistent with this Th1-skewed response, the proportion of CD3^+^CD8^+^ cytotoxic T lymphocytes increased significantly in the fusion vaccine group (5.90%), directly linking the Th1 cytokine milieu to enhanced effector T-cell expansion. Notably, the inclusion of ChXCL1 did not further augment IFN-γ or IL-12 production, indicating that EmEF1α alone serves as a potent intrinsic driver of Th1 immunity. Regarding Th2 immunity, although the fusion vaccine elicited a marginal rise in IL-4 levels, this change was not significant and suggested that ChXCL1 did not primarily act to amplify Th2 humoral immune axis. In addition, ChXCL1 significantly upregulated the immunoregulatory cytokine IL-10, which exerts dual anti-inflammatory- and tolerance-modulating functions [39]. Nevertheless, the superior overall protective efficacy conferred by the fusion vaccine is likely attributable not to modulation of any single cytokine, but rather to its integrated, multi-mechanistic immunoenhancing profile, including balanced Th1/Th2 polarization, strengthened cellular immunity, and fine-tuned regulation of inflammatory responses. This underscores the central mechanism by which ChXCL1 functions as a molecular adjuvant: achieving effective protection through holistic, coordinated immunomodulation.

The ChXCL1-EmEF1α fusion vaccine elicits a progressive and sustained increase in anti-EmEF1α IgY levels, which is a functional avian analog of mammalian IgG and a well-established serological marker of systemic adaptive immunity [40]. These IgG levels remain significantly higher than those observed in the single-component immunization group throughout the immunization regimen. Concurrently, antibody titers induced by the fusion vaccine consistently exceed those elicited by the single-component vaccine, demonstrating that ChXCL1 acts as an effective molecular adjuvant capable of potently enhancing antigen-specific humoral immunity. Moreover, the significantly elevated serum IgA levels observed in the fusion vaccine group indicate reinforcement of the mucosal immune barrier, collectively highlighting ChXCL1’s dual capacity to augment both systemic and mucosal antibody responses.

*E. maxima*, a highly pathogenic coccidian parasite that specifically targets the nutrient absorptive regions of the jejunum and ileum, induces severe growth suppression in infected hosts [41]. In our experimental challenge model, unvaccinated and challenge control chickens exhibited pronounced weight loss and elevated intestinal lesion scores. By contrast, the ChXCL1-EmEF1α fusion vaccine conferred robust protective immunity (ACI = 170), resulting in a 71.7% improvement in relative weight gain, a 66.4% reduction in oocyst shedding, and significant attenuation of intestinal lesions. These findings demonstrate that the vaccine effectively preserves intestinal structural integrity and physiological function, thereby translating immunological protection into measurable improvements in production performance and economic return [11].

## 5. Conclusions

The ChXCL1–EmEF1α fusion antigen was successfully expressed and delivered as a DNA vaccine. Immunization trials demonstrated that this construct confers robust protection against *Eimeria maxima*, as evidenced by significantly improved weight gain, markedly reduced oocyst shedding, and attenuated intestinal lesions. Protective efficacy correlated strongly with enhanced cellular immunity, including expansion of both CD4^+^ and CD8^+^ T-cell populations and a Th1-skewed cytokine profile as well as augmented humoral immune responses. These findings validate the utility of the DNA vaccine platform and underscore ChXCL1’s potential as a potent molecular adjuvant, offering a promising strategy for the development of next-generation vaccines against avian coccidiosis.

## Figures and Tables

**Table 1:** Primers used in this study.

Primer Name	Primer Sequence 5′ → 3′	Gene Bank ID
*ChXCL1*-EcoR I-F	CTTAAGGAATTCGCCACCATGGTGGCAAGCCAGAGTATGCG	NP_990377
*ChXCL1*-Xho I-R	CTCGAGTCAGTGGTGGTGGTGGTGGTGACGACGGCGGGTGGTA	
*EmEF1α*-EcoR I-F	GAATTCGCCACCATGGGTAAAGAAAAAACCCAT	XP_013336300.1
*EmEF1α*-Xho I-R	CTCGAGTCAGTGGTGGTGGTGGTGGTGTTTTTTAGCTGCGGC	
*ChXCL1-EmEF1α*-Afl II-F	CTTAAGGAATTCGCCACCATGGTGGCAAGCCAGAGTATGCG	NP_990377, and XP_013336300.1
*ChXCL1-EmEF1α*-Xho I-R	CTCGAGTCAGTGGTGGTGGTGGTGGTGTTTTTTAGCTGCGGC	

**Table 2:** Lymphocyte characteristics for each group.

Groups	CD4^+^ T (%)	CD8^+^ T (%)
PBS	4.79 ± 0.50 ^c^	2.74 ± 1.37 ^b^
pVAX1	6.71 ± 0.75 ^b^	2.52 ± 0.36 ^b^
pVAX1-ChXCL1	8.84 ± 1.71 ^b^	3.87 ± 1.03 ^ab^
pVAX1-EmEF1α	10.21 ± 1.64 ^ab^	4.81 ± 1.23 ^a^
pVAX1-ChXCL1-EmEF1α	11.76 ± 0.87 ^a^	5.58 ± 0.73 ^a^

Note: Values are expressed as mean ± SD (*n* = 6). Means in the same column with different letters were significantly different between treatment groups (*p* < 0.05).

**Table 3:** Protective efficacy of pVAX1-ChXCL1, pVAX1-EmEF1α, and pVAX1-ChXCL1-EmEF1α DNA plasmid.

Groups	Mean Lesion Score (*n* = 4)	Average Weight Gain (AWG) (*n* = 11)	Rate of Relative Body Weight Gain (RWG, %)	Average Amount of Oocyst (×10^7^) (*n* = 11)	Oocyst Reduction Rate (%)	Anticoccidial Index (ACI)
uu	0	194.53 ± 65.20 ^a^	100	0 ^e^	-	200
uc	3.66 ± 0.48 ^a^	34.6 ± 24.22 ^e^	17.8	2.53 ± 0.29 ^a^	0	111
pVAX1	2.89 ± 0.75 ^b^	46.35 ± 13.71 ^de^	23.8	2.25 ± 0.29 ^ab^	11.1	117
pVAX1-ChXCL1	2.06 ± 0.87 ^c^	91.48 ± 21.59 ^cd^	47.0	1.97 ± 0.21 ^b^	22.1	146
pVAX1-EmEF1α	1.51 ± 0.51 ^d^	107.7 ± 24.11 ^bc^	55.4	1.56 ± 0.26 ^c^	38.3	152
pVAX1-ChXCL1-EmEF1α	1.22 ± 0.65 ^d^	139.38 ± 24.41 ^b^	71.7	0.85 ± 0.11 ^d^	66.4	170

Note: Values are expressed as mean ± SD. Means in the same column with different letters were significantly different between treatment groups (*p* < 0.05).

## Data Availability

The original contributions presented in this study are included in the article. Further inquiries can be addressed to the corresponding author.

## Supplementary Materials

The following supporting information can be downloaded at https://www.mdpi.com/article/10.3390/ani16071108/s1, Figure S1. Cloning and plasmid construction of *ChXCL1*, *EmEF1α*, and *ChXCL1-EmEF1α* genes. (A–C): Amplification of the *ChXCL1*, *EmEF1α* and *ChXCL1-EmEF1α* gene. Lane M: Trans2K DNA Marker; lane 1: *ChXCL1* gene; lane 2: *EmEF1α* gene; lane 3: *ChXCL1-EmEF1α* gene; lane N: negative control group. (D): Double digestion identification of recombinant pVAX1-ChXCL1, pVAX1-EmEF1α, and pVAX1-ChXCL1-EmEF1α plasmids. Lane M: Trans2K DNA Marker; lane 1: the digested product of pVAX1-ChXCL1 by EcoR I and Xho I; lane 2: the digested product of pVAX1-EmEF1α by EcoR I and Xho I; lane 3: the digested product of pVAX1-ChXCL1-EmEF1α by Afl II and Xho I.
