# Supplementary material for: Evaluation of Immunoprotective Effects of DNA Vaccine Based on Eimeria maxima EF-1α Antigen and Chicken XCL1 Chemokine
Source: Animals (Basel). 2026 Apr 3;16(7):1108. doi: 10.3390/ani16071108 (PMC13072396; doi:10.3390/ani16071108)
Supplement: Supplementary file 1 [file animals-16-01108-s001.zip › animals-4125048-supplementary.pdf]

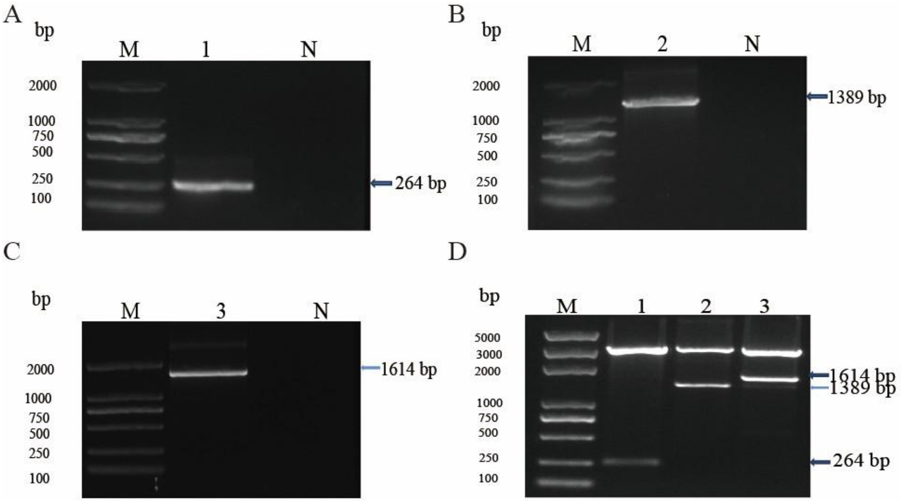

Figure S1. Cloning and plasmid construction of ChXCL1, EmEF1*α*, and ChXCL1-EmEF1*α* genes

(A-C): Amplification of the ChXCL1, EmEF1*α* and ChXCL1-EmEF1*α* gene. Lane M: Trans2K DNA Marker; lane 1: ChXCL1 gene; lane 2: EmEF1*α* gene; lane 3: ChXCL1-EmEF1*α* gene; lane N: negative control group. D: Double digestion identification of recombinant pVAX1-ChXCL1, pVAX1-EmEF1*α*, and pVAX1-ChXCL1-EmEF1*α* plasmids. Lane M: Trans2K DNA Marker; lane 1: the digested product of pVAX1-ChXCL1 by *EcoR* I and *Xho* I; lane 2: the digested product of pVAX1-EmEF1*α* by *EcoR* I and *Xho* I; lane 3: the digested product of pVAX1-ChXCL1-EmEF1*α* by *Afl* II and *Xho* I.
